# 2,2,7,7-Tetra­methyl-2,3,6,7-tetra­hydro­benzofuro[7,6-*b*]furan

**DOI:** 10.1107/S1600536810004423

**Published:** 2010-02-10

**Authors:** Xian-Fu Luo, Lin-Tao Yang, Yu Wang, Jian-Yu Zhang, Ai-Xi Hu

**Affiliations:** a College of Chemistry and Chemical Engineering, Hunan University, Changsha 410082, People’s Republic of China; b Hunan Research Institute of Chemical Industry, Changsha 410007, People’s Republic of China

## Abstract

The title compound, C_14_H_18_O_2_, was obtained as a by-product during the preparation of carbofuran phenol. The two dihydro­furan rings are in envelope conformations.

## Related literature

For chemical background and related structures, see: Xu *et al.* (2005 ▶); Li *et al.* (2009 ▶).
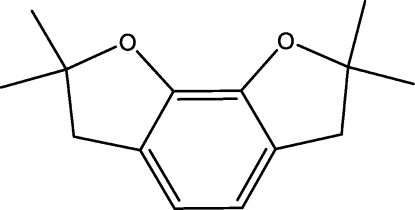


## Experimental

### 

#### Crystal data

C_14_H_18_O_2_
*M* *_r_* = 218.28
Monoclinic, 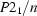

*a* = 8.7553 (6) Å
*b* = 6.0721 (4) Å
*c* = 23.2082 (17) Å
β = 92.186 (1)°
*V* = 1232.92 (15) Å^3^
*Z* = 4
Mo *K*α radiation
μ = 0.08 mm^−1^
*T* = 173 K
0.45 × 0.44 × 0.39 mm

#### Data collection

Bruker SMART 1000 CCD diffractometer
Absorption correction: multi-scan (*SADABS*; Sheldrick, 2004 ▶) *T*_min_ = 0.966, *T*_max_ = 0.971
5882 measured reflections
2662 independent reflections
1986 reflections with *I* > 2σ(*I*)
*R*_int_ = 0.022

#### Refinement

*R*[*F* ^2^ > 2σ(*F* ^2^)] = 0.045
*wR*(*F* ^2^) = 0.131
*S* = 1.04
2662 reflections
149 parameters
H-atom parameters constrained
Δρ_max_ = 0.25 e Å^−3^
Δρ_min_ = −0.18 e Å^−3^

### 

Data collection: *SMART* (Bruker, 2001 ▶); cell refinement: *SAINT-Plus* (Bruker, 2003 ▶); data reduction: *SAINT-Plus*; program(s) used to solve structure: *SHELXTL* (Sheldrick 2008 ▶); program(s) used to refine structure: *SHELXTL*; molecular graphics: *SHELXTL*; software used to prepare material for publication: *SHELXTL*.

## Supplementary Material

Crystal structure: contains datablocks I, New_Global_Publ_Block. DOI: 10.1107/S1600536810004423/bt5185sup1.cif

Structure factors: contains datablocks I. DOI: 10.1107/S1600536810004423/bt5185Isup2.hkl

Additional supplementary materials:  crystallographic information; 3D view; checkCIF report

## Figures and Tables

**Fig. 1.**
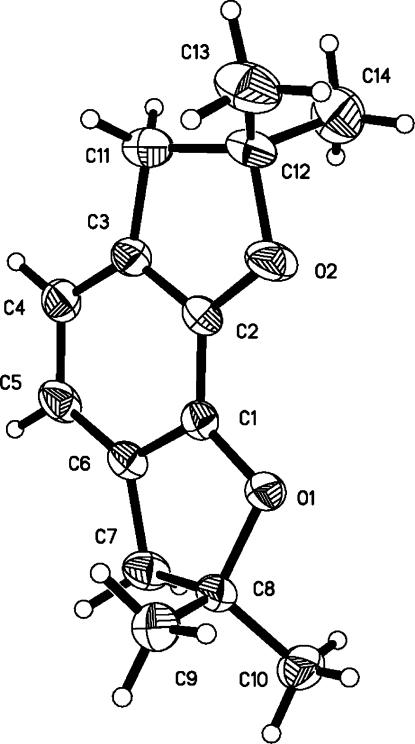
The structure of the title compound showing 30% probability displacement ellipsoids.

**Fig. 2.**
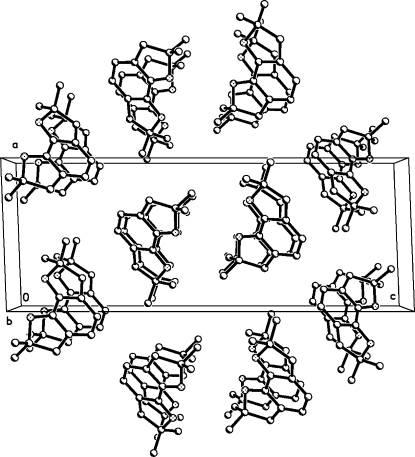
A packing diagram for the title compound. H atoms bonded to C atoms have been omitted for clarity.

## supplementary crystallographic information

### Comment

Carbofuran phenol (systematic name: 2,2-dimethy-2,3-dihydrobenzofuran-7-ol) is an important intermediate to prepare Carbofuran (Xu *et al.*, 2005), Carbosulfan, Benfuracarb, Furathiocarb and other large tonnage carbamate pesticides. It also can be used as pharmaceutical intermediate, as a high value-added fine chemical product. The title compound, 2,2,7,7-tetramethyl-2,3,6,7-tetrahydrobenzofuro[7,6-b]furan,   was obtained as a byproduct during the preparation of carbofuran phenol.

### Experimental

After distillation of carbofuran phenol, the fraction at 433.15 K(3.33 K Pa) was cooled to room temperature, then the precipitate was emerged. The solid was purified by recrystallization from saturated ethyl acetate solution, giving the title compound as a colourless crystalline solid. Single crystals suitable for X-ray diffraction were obtained by slow evaporation of an ethyl acetate solution at room temperature over a period of ten days. The identity of the title compound was confirmed by NMR and GC—MS spectroscopy.

### Refinement

All H atoms were placed in calculated positions, with C—H ranging from  0.95 Å to  0.99 Å  and with *U*iso(H) = 1.5U_eq_(C_methyl_) or *U*iso(H) = 1.2U_eq_(C). The methyl groups were allowed to rotate but not to tip.

### Crystal data

**Table:**

C_14_H_18_O_2_	*F*(000) = 472
*M**_r_* = 218.28	*D*_x_ = 1.176 Mg m^−^^3^
Monoclinic, *P*2_1_/*n*	Melting point: 344.25 K
Hall symbol: -P 2yn	Mo *K*α radiation, λ = 0.71073 Å
*a* = 8.7553 (6) Å	Cell parameters from 2852 reflections
*b* = 6.0721 (4) Å	θ = 2.5–27.0°
*c* = 23.2082 (17) Å	µ = 0.08 mm^−^^1^
β = 92.186 (1)°	*T* = 173 K
*V* = 1232.92 (15) Å^3^	Block, colourless
*Z* = 4	0.45 × 0.44 × 0.39 mm

### Data collection

**Table:**

Bruker SMART 1000 CCD diffractometer	2662 independent reflections
Radiation source: fine-focus sealed tube	1986 reflections with *I* > 2σ(*I*)
graphite	*R*_int_ = 0.022
ω scans	θ_max_ = 27.0°, θ_min_ = 1.8°
Absorption correction: multi-scan (*SADABS*; Sheldrick, 2004)	*h* = −11→4
*T*_min_ = 0.966, *T*_max_ = 0.971	*k* = −7→7
5882 measured reflections	*l* = −27→29

### Refinement

**Table:**

Refinement on *F*^2^	Primary atom site location: structure-invariant direct methods
Least-squares matrix: full	Secondary atom site location: difference Fourier map
*R*[*F*^2^ > 2σ(*F*^2^)] = 0.045	Hydrogen site location: inferred from neighbouring sites
*wR*(*F*^2^) = 0.131	H-atom parameters constrained
*S* = 1.04	*w* = 1/[σ^2^(*F*_o_^2^) + (0.0698*P*)^2^ + 0.2701*P*] where *P* = (*F*_o_^2^ + 2*F*_c_^2^)/3
2662 reflections	(Δ/σ)_max_ < 0.001
149 parameters	Δρ_max_ = 0.25 e Å^−^^3^
0 restraints	Δρ_min_ = −0.17 e Å^−^^3^

### Special details

**Table:**

Experimental. ^1^H NMR(300MHz, CDCl_3_), delta: 1.49(s, 12H, CH_3_); 2.99(s, 4H, CH_2_); 6.63(s, 2H, C_6_H_2_). GC-MS(m/z): 218, 203, 185, 175, 161.
Geometry. All esds (except the esd in the dihedral angle between two l.s. planes) are estimated using the full covariance matrix. The cell esds are taken into account individually in the estimation of esds in distances, angles and torsion angles; correlations between esds in cell parameters are only used when they are defined by crystal symmetry. An approximate (isotropic) treatment of cell esds is used for estimating esds involving l.s. planes.
Refinement. Refinement of *F*^2^ against ALL reflections. The weighted *R*-factor wR and goodness of fit *S* are based on *F*^2^, conventional *R*-factors *R* are based on *F*, with *F* set to zero for negative *F*^2^. The threshold expression of *F*^2^ > σ(*F*^2^) is used only for calculating *R*-factors(gt) etc. and is not relevant to the choice of reflections for refinement. *R*-factors based on *F*^2^ are statistically about twice as large as those based on *F*, and *R*- factors based on ALL data will be even larger.

### Fractional atomic coordinates and isotropic or equivalent isotropic displacement parameters (Å^2^)

**Table:**

	*x*	*y*	*z*	*U*_iso_*/*U*_eq_	
C1	−0.00729 (15)	0.4208 (2)	0.12566 (5)	0.0263 (3)	
C2	0.10104 (15)	0.2615 (2)	0.13845 (6)	0.0278 (3)	
C3	0.09122 (16)	0.1360 (2)	0.18790 (6)	0.0303 (3)	
C4	−0.02597 (17)	0.1695 (3)	0.22541 (6)	0.0362 (4)	
H4	−0.0320	0.0825	0.2593	0.043*	
C5	−0.13474 (16)	0.3316 (3)	0.21305 (6)	0.0370 (4)	
H5	−0.2154	0.3568	0.2385	0.044*	
C6	−0.12429 (15)	0.4558 (2)	0.16334 (6)	0.0284 (3)	
C7	−0.22006 (17)	0.6411 (3)	0.13820 (6)	0.0344 (4)	
H7A	−0.1985	0.7813	0.1587	0.041*	
H7B	−0.3305	0.6076	0.1397	0.041*	
C8	−0.16891 (15)	0.6516 (2)	0.07551 (6)	0.0288 (3)	
C9	−0.26730 (18)	0.5101 (3)	0.03545 (7)	0.0365 (4)	
H9A	−0.2232	0.5065	−0.0027	0.055*	
H9B	−0.3707	0.5718	0.0322	0.055*	
H9C	−0.2719	0.3601	0.0509	0.055*	
C10	−0.1533 (2)	0.8823 (3)	0.05219 (8)	0.0426 (4)	
H10A	−0.0817	0.9660	0.0772	0.064*	
H10B	−0.2534	0.9549	0.0511	0.064*	
H10C	−0.1148	0.8758	0.0131	0.064*	
C11	0.21711 (19)	−0.0321 (3)	0.18774 (7)	0.0436 (4)	
H11A	0.1756	−0.1821	0.1814	0.052*	
H11B	0.2783	−0.0295	0.2245	0.052*	
C12	0.31387 (17)	0.0417 (3)	0.13672 (7)	0.0361 (4)	
C13	0.3435 (2)	−0.1428 (3)	0.09508 (9)	0.0541 (5)	
H13A	0.2458	−0.2021	0.0800	0.081*	
H13B	0.4015	−0.2597	0.1151	0.081*	
H13C	0.4022	−0.0865	0.0631	0.081*	
C14	0.4577 (2)	0.1601 (3)	0.15651 (8)	0.0552 (5)	
H14A	0.5094	0.2176	0.1229	0.083*	
H14B	0.5258	0.0573	0.1775	0.083*	
H14C	0.4317	0.2822	0.1819	0.083*	
O1	−0.01419 (11)	0.55235 (18)	0.07800 (4)	0.0333 (3)	
O2	0.21901 (12)	0.2053 (2)	0.10432 (4)	0.0408 (3)	

### Atomic displacement parameters (Å^2^)

**Table:**

	*U*^11^	*U*^22^	*U*^33^	*U*^12^	*U*^13^	*U*^23^
C1	0.0245 (7)	0.0291 (7)	0.0254 (7)	−0.0008 (5)	0.0020 (5)	−0.0003 (5)
C2	0.0230 (7)	0.0322 (8)	0.0282 (7)	0.0018 (6)	0.0028 (5)	−0.0035 (6)
C3	0.0259 (7)	0.0317 (8)	0.0329 (7)	−0.0001 (6)	−0.0033 (5)	0.0010 (6)
C4	0.0309 (8)	0.0484 (9)	0.0295 (7)	−0.0024 (7)	0.0010 (6)	0.0120 (7)
C5	0.0260 (7)	0.0565 (10)	0.0288 (7)	0.0044 (7)	0.0065 (5)	0.0053 (7)
C6	0.0241 (7)	0.0341 (8)	0.0272 (7)	0.0020 (6)	0.0015 (5)	−0.0024 (6)
C7	0.0321 (8)	0.0383 (8)	0.0330 (8)	0.0093 (6)	0.0025 (6)	−0.0032 (6)
C8	0.0242 (7)	0.0285 (7)	0.0339 (7)	0.0040 (6)	0.0018 (5)	0.0005 (6)
C9	0.0379 (8)	0.0350 (8)	0.0364 (8)	−0.0012 (7)	−0.0006 (6)	−0.0028 (6)
C10	0.0421 (9)	0.0302 (8)	0.0547 (10)	−0.0037 (7)	−0.0068 (7)	0.0061 (7)
C11	0.0390 (9)	0.0416 (9)	0.0501 (10)	0.0108 (7)	0.0015 (7)	0.0082 (8)
C12	0.0266 (7)	0.0388 (9)	0.0425 (9)	0.0087 (6)	−0.0041 (6)	−0.0028 (7)
C13	0.0450 (10)	0.0521 (11)	0.0649 (12)	0.0124 (8)	−0.0022 (8)	−0.0167 (9)
C14	0.0455 (10)	0.0595 (12)	0.0595 (11)	−0.0107 (9)	−0.0124 (9)	0.0016 (9)
O1	0.0268 (5)	0.0397 (6)	0.0339 (5)	0.0067 (4)	0.0074 (4)	0.0099 (4)
O2	0.0341 (6)	0.0526 (7)	0.0365 (6)	0.0184 (5)	0.0103 (4)	0.0057 (5)

### Geometric parameters (Å, °)

**Table:**

C1—O1	1.3639 (16)	C9—H9A	0.9800
C1—C2	1.3792 (19)	C9—H9B	0.9800
C1—C6	1.3882 (19)	C9—H9C	0.9800
C2—O2	1.3685 (16)	C10—H10A	0.9800
C2—C3	1.383 (2)	C10—H10B	0.9800
C3—C4	1.386 (2)	C10—H10C	0.9800
C3—C11	1.502 (2)	C11—C12	1.548 (2)
C4—C5	1.392 (2)	C11—H11A	0.9900
C4—H4	0.9500	C11—H11B	0.9900
C5—C6	1.384 (2)	C12—O2	1.4812 (18)
C5—H5	0.9500	C12—C14	1.507 (2)
C6—C7	1.508 (2)	C12—C13	1.509 (2)
C7—C8	1.540 (2)	C13—H13A	0.9800
C7—H7A	0.9900	C13—H13B	0.9800
C7—H7B	0.9900	C13—H13C	0.9800
C8—O1	1.4816 (16)	C14—H14A	0.9800
C8—C10	1.510 (2)	C14—H14B	0.9800
C8—C9	1.511 (2)	C14—H14C	0.9800
			
O1—C1—C2	126.45 (12)	H9A—C9—H9C	109.5
O1—C1—C6	114.22 (12)	H9B—C9—H9C	109.5
C2—C1—C6	119.32 (13)	C8—C10—H10A	109.5
O2—C2—C1	125.32 (13)	C8—C10—H10B	109.5
O2—C2—C3	114.54 (12)	H10A—C10—H10B	109.5
C1—C2—C3	120.07 (13)	C8—C10—H10C	109.5
C2—C3—C4	120.76 (13)	H10A—C10—H10C	109.5
C2—C3—C11	107.66 (13)	H10B—C10—H10C	109.5
C4—C3—C11	131.47 (14)	C3—C11—C12	103.19 (12)
C3—C4—C5	119.45 (13)	C3—C11—H11A	111.1
C3—C4—H4	120.3	C12—C11—H11A	111.1
C5—C4—H4	120.3	C3—C11—H11B	111.1
C6—C5—C4	119.37 (13)	C12—C11—H11B	111.1
C6—C5—H5	120.3	H11A—C11—H11B	109.1
C4—C5—H5	120.3	O2—C12—C14	106.33 (13)
C5—C6—C1	121.03 (13)	O2—C12—C13	106.23 (13)
C5—C6—C7	132.51 (13)	C14—C12—C13	112.80 (15)
C1—C6—C7	106.46 (12)	O2—C12—C11	105.66 (11)
C6—C7—C8	102.64 (11)	C14—C12—C11	112.36 (14)
C6—C7—H7A	111.2	C13—C12—C11	112.79 (15)
C8—C7—H7A	111.2	C12—C13—H13A	109.5
C6—C7—H7B	111.2	C12—C13—H13B	109.5
C8—C7—H7B	111.2	H13A—C13—H13B	109.5
H7A—C7—H7B	109.2	C12—C13—H13C	109.5
O1—C8—C10	107.24 (12)	H13A—C13—H13C	109.5
O1—C8—C9	106.96 (11)	H13B—C13—H13C	109.5
C10—C8—C9	111.36 (12)	C12—C14—H14A	109.5
O1—C8—C7	104.17 (10)	C12—C14—H14B	109.5
C10—C8—C7	114.23 (13)	H14A—C14—H14B	109.5
C9—C8—C7	112.23 (12)	C12—C14—H14C	109.5
C8—C9—H9A	109.5	H14A—C14—H14C	109.5
C8—C9—H9B	109.5	H14B—C14—H14C	109.5
H9A—C9—H9B	109.5	C1—O1—C8	106.37 (10)
C8—C9—H9C	109.5	C2—O2—C12	107.10 (11)
			
O1—C1—C2—O2	−1.5 (2)	C6—C7—C8—O1	−23.68 (14)
C6—C1—C2—O2	177.75 (13)	C6—C7—C8—C10	−140.34 (13)
O1—C1—C2—C3	−178.26 (13)	C6—C7—C8—C9	91.68 (14)
C6—C1—C2—C3	1.0 (2)	C2—C3—C11—C12	8.91 (17)
O2—C2—C3—C4	−177.69 (13)	C4—C3—C11—C12	−174.97 (15)
C1—C2—C3—C4	−0.6 (2)	C3—C11—C12—O2	−13.11 (16)
O2—C2—C3—C11	−1.07 (18)	C3—C11—C12—C14	102.42 (16)
C1—C2—C3—C11	176.00 (13)	C3—C11—C12—C13	−128.73 (14)
C2—C3—C4—C5	−0.1 (2)	C2—C1—O1—C8	165.26 (13)
C11—C3—C4—C5	−175.75 (16)	C6—C1—O1—C8	−14.05 (15)
C3—C4—C5—C6	0.3 (2)	C10—C8—O1—C1	144.79 (12)
C4—C5—C6—C1	0.1 (2)	C9—C8—O1—C1	−95.65 (13)
C4—C5—C6—C7	−179.27 (15)	C7—C8—O1—C1	23.36 (14)
O1—C1—C6—C5	178.59 (13)	C1—C2—O2—C12	175.32 (13)
C2—C1—C6—C5	−0.8 (2)	C3—C2—O2—C12	−7.79 (16)
O1—C1—C6—C7	−1.89 (16)	C14—C12—O2—C2	−106.65 (15)
C2—C1—C6—C7	178.75 (12)	C13—C12—O2—C2	132.97 (14)
C5—C6—C7—C8	−164.44 (16)	C11—C12—O2—C2	12.94 (16)
C1—C6—C7—C8	16.11 (15)		
